# Impact of the COVID-19 pandemic on imaging case volumes in King Abdullah University Hospitals (KAUH)

**DOI:** 10.3389/fmed.2023.1103083

**Published:** 2023-02-09

**Authors:** Maha Gharaibeh, Eyhab El-Obeid, Ruba Khasawneh, Musaab Karrar, Mohamed Salman, Ahmad Farah, Sammah Ahmmed, Mamoon Al-Omari, Mwaffaq Elheis, Laith Abualigah

**Affiliations:** ^1^Faculty of Medicine, Department of Diagnostic and Interventional Radiology, Jordan University of Science and Technology, Irbid, Jordan; ^2^Faculty of Medicine, Department of Diagnostic Radiology, Omdurman Islamic University, Omdurman, Sudan; ^3^Faculty of Medicine, Department of Emergency, Jordan University of Science and Technology, Irbid, Jordan; ^4^Faculty of Medicine, Department of Emergency, Omdurman Islamic University, Omdurman, Sudan; ^5^Prince Hussein Bin Abdullah Faculty for Information Technology, Computer Science Department, Al al-Bayt University, Mafraq, Jordan; ^6^Hourani Center for Applied Scientific Research, Al-Ahliyya Amman University, Amman, Jordan; ^7^Faculty of Information Technology, Middle East University, Amman, Jordan; ^8^Applied Science Research Center, Applied Science Private University, Amman, Jordan; ^9^School of Computer Sciences, Universiti Sains Malaysia, Penang, Malaysia

**Keywords:** COVID-19, radiological modality types, imaging volume, patient service locations, images

## Abstract

**Objective:**

COVID-19 has an increased burden on the delivery of services because the measures taken by the governments forced hospitals to cancel most of their elective procedures and led to the shutting down of outpatient clinics. This study aimed to evaluate the impact COVID-19 pandemic on the volume of radiology exams based on patient service locations and imaging modality in the North of Jordan.

**Methods:**

The imaging case volumes that were performed at the King Abdullah University Hospital (KAUH), Jordan, from 1 January 2020 to 8 May 2020, were retrospectively collected and compared to those from 1 January 2019 to 28 May 2019, to determine the impact of the pandemic of COVID-19 on the volume of radiological examinations. The 2020 study period was chosen to cover the peak of COVID-19 cases and to record the effects on imaging case volumes.

**Results:**

A total of 46,194 imaging case volumes were performed at our tertiary center in 2020 compared to 65,441 imaging cases in 2019. Overall, the imaging case volume in 2020 decreased by 29.4% relative to the same period in 2019. The imaging case volumes decreased for all imaging modalities relative to 2019. The number of nuclear images showed the highest decline (41.0%) in 2020, followed by the number of ultrasounds (33.2%). Interventional radiology was the least affected imaging modality by this decline, with about a 22.9% decline.

**Conclusion:**

The number of imaging case volumes decreased significantly during the COVID-19 pandemic and its associated lockdown. The outpatient service location was the most affected by this decline. Effective strategies must be adopted to avoid the aforementioned effect on the healthcare system in future pandemics.

## Introduction

The World Health Organization (WHO) declared the COVID-19 pandemic in March 2020 (1, 2). Around the world, 3,502,956 cases and 245,081 deaths have been reported as of 3 May 2020 (3). The majority of countries around the world adopted various measures to stop the virus’s spread (4, 5). In Jordan, a country with a population of about 10 million, on 2 March 2020, the Ministry of Health announced the first case. In response to the virus’s quick spread in Jordan’s neighboring countries and worldwide, the authorities closed the borders and airports on 14 March 2020, stopped schools, and restricted public gatherings. The government declared a total lockdown on 17 March 2020, which was hailed as one of the strictest measures in the history of the world. This measure lasted for about 6 weeks and included a ban on car use, except for health service providers and employees of important sectors.

However, during this time, people were permitted to walk to nearby stores to buy groceries from 10 a.m. to 6 p.m. On the healthcare side, all public and private clinics were closed, all scheduled operations and non-urgent outpatient visits were delayed, and only emergency operations were performed to save expenses on medical care services for patients with COVID-19 (6). During that time, the Jordan government’s policy was to admit all COVID-19-positive patients to the hospital even if they did not have any symptoms (7).

Moreover, elective imaging services were stopped to decrease the virus’s transmission (8). This decision had a significant impact on hospitals’ level of financial revenue. On the other hand, hospitals have redirected their financial resources to deal with this pandemic, prepare isolation departments, train staff, and purchase PPE (9, 10). Despite the measures taken, the city of Irbid, where this study was conducted, has turned into a hotbed for the spread of the COVID-19 virus in Jordan (9). The economic burdens on Jordan and the need for medical resources have increased. Anecdotal articles suggest that the number of imaging cases has decreased by 50–70% during the pandemic (11). In a New York City study, the researchers analyzed medical imaging cases from numerous institutions between 1 January 2019, Pre- COVID-19, and 18 April 2020, post-COVID-19. According to this report, there has been a 28% overall decline in imaging volume (6). Another study in Germany showed that overall case volumes decreased by an average of 41% during the shutdown compared to the control period (12). According to a study by Alelyani et al. performed in the Saudi Arabian province of Aseer, the COVID-19 pandemic impacted the number of medical imaging cases there, with a reported decrease of 21.27% in 2020 compared to 2019 (13).

This study aimed to determine the decline in imaging case volumes caused by the COVID-19 pandemic and how it affected different patient service locations and types of imaging modality (6). This study looked back at the data of a large healthcare system to examine the weekly imaging volumes from different patient service locations (emergency department, inpatient, and outpatient) and modality types (x-ray, mammography, CT, MRI, ultrasound, interventional radiology, and nuclear medicine) in 2020 and 2019. The data was separated to compare the pre-COVID-19 period (weeks 1–9) and post-COVID-19 period (weeks 10–16), and the mean weekly volumes were compared using independent-samples *t*-tests.

This study aimed to examine the actual impact of the COVID-19 pandemic on radiology practices, by looking at changes in imaging utilization and revenue (14). Using data from three high-volume hospitals, imaging performed between 8 March and 30 April 2020 was categorized as taking place during the COVID-19 healthcare crisis. The volume of imaging during this time was compared to the normal practice expected volume, which was calculated using data from the 10 weeks prior. The study examined total imaging utilization by patient setting (outpatient, inpatient, and emergency) and imaging modality (X-ray, CT, Mammography, MRI, Nuclear Medicine/PET, and US) and also collected revenue information from the hospital billing system.

This study aimed to evaluate the effect of the COVID-19 pandemic on imaging volumes for radiology residents, in relation to their training year and imaging modality (15). Radiology resident reports were collected during specific pre- and during-pandemic periods, then imaging case volumes were analyzed to determine the change in volume by training level and imaging modality. The study also looked at the number of days that residents were assigned to work outside of the normal reading room.

Nevertheless, there is not much published real-world data to support these numbers. The pandemic has an increased burden on the delivery of services because the measures taken by the government forced hospitals to cancel most of their elective procedures and led to the shutting down of outpatient clinics. This study aimed to evaluate the impact COVID-19 pandemic on the volume of radiological exams based on patient service locations and imaging modality in the North of Jordan. To our knowledge, no published study has examined how the pandemic affected Jordan’s radiology departments. This study may contribute to developing plans for the crisis-related gradual return and continued operation of radiology departments. It will offer relevant information for future evidence-based decisions about similar public health emergencies.

## Materials and methods

The imaging case volumes that were performed at the King Abdullah University Hospital (KAUH), Jordan, from 1 January 2020 to 28 May 2020, were retrospectively collected and compared to those from 1 January 2019 to 28 May 2019, to determine the impact of the pandemic of COVID-19 on the volume of radiological examinations. The 2020 study period was chosen to cover the peak of COVID-19 cases and to record the effects on imaging case volumes.

The administrators of the King Abdullah University Hospital’s picture archiving and communication system (PACS) provided the total volume of imaging cases for each period. The imaging cases were further divided into categories based on the patients’ service setting (outpatient, inpatient, or emergency department) and the type of imaging modality (X-rays, interventional radiology, ultrasound, mammogram, nuclear medicine, MRI, and CT).

The radiology department in our hospital did not note any significant changes in the imaging devices or staff during the study period that could have impacted the number of radiological procedures.

The Declaration of Helsinki was followed in conducting this retrospective research. Ethical approval was obtained from the King Abdulla University Hospital Institutional Review Board (IRB) (approved on 18/10/2020, number: 22/136/2020). Our local IRB waived the requirement of obtaining a signed consent form for the subjects included in this study.

Data were described using IBM SPSS version 23. The total number of imaging case volumes was calculated for each radiological modality and patient service location. The total number of imaging case volumes per radiology modality and patient service location for the first 20 weeks of 2020 were aggregated for each month and plotted using a line graph. Percent of change in the number of imaging case volumes between the two periods was calculated by subtracting the number of images in the period 1 January 2020–28 May 2020 from that in the period 1 January 2019–28 May 2019 and dividing difference by the number of images for the period 1 January 2019–28 May 2019 (multiplied by 100%).

The COVID-19 pandemic has had a significant impact on imaging case volumes in hospitals. Due to social distancing measures and a decrease in overall hospital activity, many imaging departments have seen a decrease in case volumes. This has been particularly true for elective procedures, which have been put on hold to prioritize urgent and emergent cases related to COVID-19. This has led to a decrease in revenue for many imaging departments, as well as a disruption in the education of radiology residents due to reduced imaging volumes. In addition to the decrease in case volumes, the pandemic has also led to changes in the composition of imaging volumes. For example, there has been an increase in the use of telemedicine and remote monitoring, as well as a shift in focus toward imaging modalities that are less likely to spread the virus, such as MRI and ultrasound. Overall, the COVID-19 pandemic has greatly impacted the functioning of imaging departments in hospitals. It has led to a decrease in case volumes and revenue, as well as changes in the composition of imaging volumes. These effects are likely to be felt for some time, and hospitals will need to adapt and find new ways to maintain revenue and support the education of radiology residents in the face of these challenges.

## Results

A total of 46,194 imaging case volumes were performed at our tertiary center in the first 20 weeks of 2020 compared to a total of 65,441 imaging case volumes in the first 20 weeks of 2020. Overall, the imaging case volume in the first 20 weeks of 2020 decreased by 29.4% relative to that in the first 20 weeks of 2019. The percent change in imaging case volumes between the two periods (1 January 2020–28 May 2020, vs. 1 January 2019–28 May 2019) is stratified according to the radiology modality and patients’ service location (Table 1).

**TABLE 1:** The change in imaging case volumes between the two study periods (1 January 2020–28 May 2020, vs. 1 January 2019–28 May 2019) according to the radiology modality and patients’ service location.

Category	Period		
	1 January 2019–28 May 2019	1 January 2020–28 May 2020	% Change
**Radiology modality**			
Computerized tomography (CT)	13,267	9,286	−30.0%
Magnetic resonance imaging (MRI)	7,930	5,832	−26.5%
X-ray	33,912	24,253	−28.5%
Interventional radiology	903	696	−22.9%
Ultrasound (US)	6,585	4,397	−33.2%
Mammogram	576	391	−32.1%
Nuclear medicine	2,268	1,339	−41.0%
Total	65,441	46,194	−29.4%
**Service location**			
Emergency room	15,949	12,810	−19.7%
Inpatient	25,460	19,672	−22.7%
Outpatient	24,035	13,712	−42.9%

The imaging case volumes in the first 20 weeks of 2020 decreased for all imaging modalities relative to that in the same period of 2019. The number of nuclear images showed the highest decline (41.0%) in 2020, followed by the number of ultrasounds (33.2%). Interventional radiology was the least affected imaging modality by this decline, with about a 22.9% decline. Compared to the same period in 2019, the outpatient service location’s imaging case volumes for weeks 1 through 20 in 2020 exhibited the most noticeable decline (42.9%). The percent of the decrease in the imaging case volumes in inpatient service locations was 22.7%.

**FIGURE 1:**
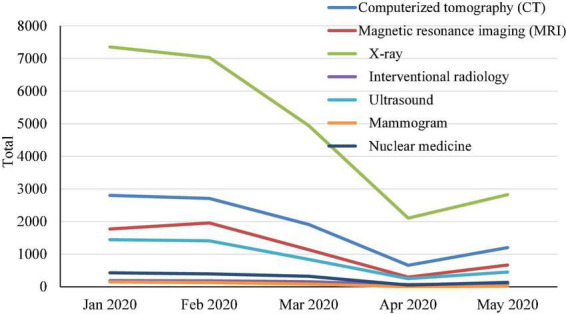
The change in imaging case volumes over time in January–May 2020 stratified by imaging modality.

**FIGURE 2:**
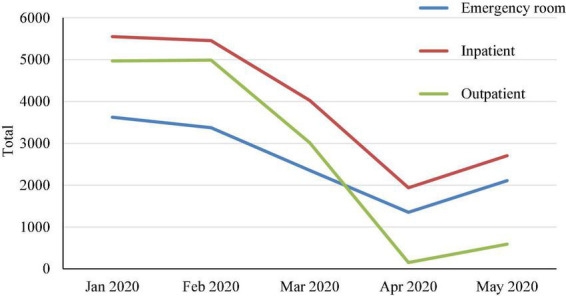
The change in imaging case volumes over time in January–May 2020 stratified by patient service location.

Figures 1, 2 outline the change in imaging case volumes over January–May 2020, stratified by imaging modality and patient service location. The imaging case volumes markedly declined in March 2020, the month of the strict lockdown in the country. The imaging case volumes continued to decline in April 2020, after which the imaging case volumes started to increase. Figure 2 shows that this monthly decline affected the outpatient service location the most. There was a gradual increase in imaging case volumes per imaging modality and patient service location in the subsequent months during COVID-19. Still, they never reached the imaging case volumes in pre-COVID-19 or in the same time frame for 2019.

## Discussion

The first epidemic in Jordan occurred in Irbid, where our institute King Abdullah University Hospital, is located. About 150 patients were affected by the end of March. This spread resulted in restricting the ban in Irbid and isolating it from the rest of the governorates. The results from our study revealed a reduction of 29.4% in the number of all radiological procedures during the COVID-19 period compared with the same time frame in 2019. The findings mentioned above are less than the anecdotal evidence indicating the imaging practice will experience a 50–70% drop in radiological imaging for at least three to 4 months, based on the COVID-19 severity in the territory. The COVID-19 pandemic’s effects on the number of medical imaging cases in the Aseer region were examined by Alelyani et al. They reported similar results of a reduction in the overall imaging case volume by 21.27% during the COVID-19 period compared with 2019 (13). Another study by Naidich et al. in New York during the COVID-19 epidemic revealed a drop of roughly 28% in the overall number of imaging (6). There was a drop of 26.45%, according to a different study done in occupational land, where the number of new cases and deaths was higher than in Jordan, and the MRI procedures fell by 47.5% during the first wave (16).

In contrast, a study by Parikh et al. revealed a 55% imaging volume loss during the COVID-19 pandemic (14). The decline in the total volume of radiological imaging in Jordan is attributed to several factors, including the population’s fear of COVID-19, strict Jordanian government actions taken to restrict the virus spread, and the general curfew, which was referred to as one of the harshest regulations in the entire world.

The epidemic led to a significant decrease in imaging according to the modality type. The most affected modalities were nuclear medicine (41%) and ultrasound (33.2%), in decreasing order, followed by mammogram (32.1%), CT (30%), X-ray (28.5%), MRI (26.6%), and interventional radiology (22.9%). Naidich et al. reported a great decline in nuclear medicine by (27.36%), then in decreasing order followed by MRI (22.71%), interventional radiology (22.64%), Mammography (21.56%), ultrasound (19.61%), CT (14.26%), and x-ray (6.87%) (5). According to our data, the decrease is primarily attributable to a decline in outpatient volumes, and the most affected modalities are outpatient-driven imaging, like nuclear medicine.

During the COVID-19 pandemic, a decline in imaging volumes was expected, especially for outpatients. Our study’s drop in imaging volume varied according to the different service locations. In our study, the most decline was seen in the outpatient (42.9%), followed by inpatient (22.7%), with the least affected department being the emergency (19.7%). A previous study revealed a different result, with the outpatient settings experiencing the greatest reduction by 24.8%, emergency departments by 10.7%, and inpatient settings by 7.43%. Another study by Schwertner et al. discovered that outpatient examinations decreased to 38.7% of the pre–COVID-19 volume, emergency department examinations to 63%, and inpatient examinations to 73% of the pre–COVID-19 volume (17). We explain that emergency patients were not affected by the lockdown to the same degree as patients from other departments. The Jordanian government put a system for transporting patients to hospitals using civil defense ambulances. Any patient who feels symptoms that indicate the need to go to the emergency room contacts the Civil Defense, which transports the patient free of charge to the nearest emergency room. This was in line with another study that found no change in stroke admissions during the early weeks of the epidemic in the ED (18).

The trend data showed a steep decline in the number of imaging case volumes observed in March 2020, which labels the beginning of the strict lockdown in Jordan. The imaging case volumes continued to decline in April 2020, after which the imaging case volumes started to increase (Figure 2). The number of imaging cases per modality and patient service location increased gradually during COVID-19. However, they never reached the numbers from the Pre-COVID-19 period or the same period in 2019.

Our hospital is accredited as a referral hospital for the Northern Jordan Region. Despite the strict measures to stop the disease from spreading, the medical need for treatment, and radiological procedures to aid in the diagnosis of COVID-19, the number of patients gradually increased in emergency and those admitted for treatment of COVID-19 disease. According to the author, this study is the first of its kind in Jordan and in developing countries. This type of study plays a crucial role in assessing how the radiology practice was affected in developing countries with a shortage of resources.

## Limitations and further studies

The study is limited by its retrospective design. The economic effects of this decline in imaging case volumes and the effects on the radiology residency program were not investigated. These limitations could serve as an avenue for future research.

## Conclusion

The number of imaging case volumes decreased significantly during the COVID-19 pandemic and its associated lockdown. The outpatient service location was the most affected by this decline. Effective strategies must be adopted to avoid the aforementioned effect on the healthcare system in future pandemics. Concluding remarks are as follows.

• According to this report, the total imaging volume across all patient care locations and imaging modalities decreased by 29.4% during the COVID-19 pandemic.
• The rate of imaging volume deterioration varied depending on the modality type, with the most significant decline observed in nuclear medicine (41%), followed in descending order by ultrasound (33.2), mammogram (32.1%), CT (30%), X-ray (28.5%), MRI (26.6%), and interventional radiology (22.9%) showing the highest declines.
• The rate of decline in imaging volume also varied by location, with outpatient settings experiencing the most significant reduction (42.9%), followed by inpatient settings (22.7%), and the emergency room (19.7%).
• The findings from this study offer real-world data to guide radiology practices toward evidence-based decisions as they get ready to reduce the COVID-19 pandemic’s impact on imaging case volumes and start developing transition strategies for the recovery period.

## Data availability statement

The raw data supporting the conclusions of this article will be made available by the authors, without undue reservation.

## Ethics statement

The King Abdulla University Hospital Institutional Review Board (IRB) gave their approval to the study protocol (approved on 18/10/2020, number: 22/136/2020). Our local IRB waived the requirement of obtaining a signed consent form for the subjects included in this study.

## Author contributions

MG and E-EO: study design. MS, AF, EE-O, and MK: data collection. MG, EE-O, RK, MK, MS, AF, SA, and MA-O: data interpretation. MG, MK, MS, and AF: data analysis. MG, EE-O, RK, MK, SA, and MA-O: literature search. MG, EE-O, RK, SA, ME, and LA: writing of the manuscript. MG, EE-O, RK, MA-O, SA, ME, and LA: writing—review and editing. All authors contributed to the article and approved the submitted version.
